# Microbial community changes correlate with impaired host fitness of *Aurelia aurita* after environmental challenge

**DOI:** 10.1186/s42523-023-00266-4

**Published:** 2023-09-21

**Authors:** Nicole Pinnow, Cynthia M. Chibani, Simon Güllert, Nancy Weiland-Bräuer

**Affiliations:** 1 General Microbiology, Kiel University, Am Botanischen Garten 1-9, 24118 Kiel, Germany; 2 Current address: Sysmex Inostics GmbH, Falkenried 88, 20251 Hamburg, Germany

**Keywords:** Metaorganism, *Aurelia aurita*, Environment, Climate change, Microbiome, Acclimatization, Salinity, Temperature, Future ocean

## Abstract

Climate change globally endangers certain marine species, but at the same time, such changes may promote species that can tolerate and adapt to varying environmental conditions. Such acclimatization can be accompanied or possibly even be enabled by a host’s microbiome; however, few studies have so far directly addressed this process. Here we show that acute, individual rises in seawater temperature and salinity to sub-lethal levels diminished host fitness of the benthic *Aurelia aurita* polyp, demonstrated by up to 34% reduced survival rate, shrinking of the animals, and almost halted asexual reproduction. Changes in the fitness of the polyps to environmental stressors coincided with microbiome changes, mainly within the phyla Proteobacteria and Bacteroidota. The absence of bacteria amplified these effects, pointing to the benefit of a balanced microbiota to cope with a changing environment. In a future ocean scenario, mimicked by a combined but milder rise of temperature and salinity, the fitness of polyps was severely less impaired, together with condition-specific changes in the microbiome composition. Our results show that the effects on host fitness correlate with the strength of environmental stress, while salt-conveyed thermotolerance might be involved. Further, a specific, balanced microbiome of *A. aurita* polyps supports the host’s acclimatization. Microbiomes may provide a means for acclimatization, and microbiome flexibility can be a fundamental strategy for marine animals to adapt to future ocean scenarios and maintain biodiversity and ecosystem functioning.

**Supplementary Information:**

The online version contains supplementary material available at 10.1186/s42523-023-00266-4.

## Introduction

It is widely recognized that marine ecosystems are under threat [1]. Ocean acidification, the global increase in sea surface temperature, and changes in salinity, along with overfishing, eutrophication, sedimentation, and pollution, endanger marine species globally [2–4]. At the same time, marine organisms adjust and cope with changing environments [5–8]. Eco-physiological studies typically focus on solitary macroorganisms or interactions among them [9, 10], such as competition (e.g., acidification influencing turf algae-kelp interactions) and predation (e.g., warming leading to kelp grazing by range-expanding herbivorous fishes) [11–13].

In contrast, less is known about the impact of ocean climate change on interactions between macro- and microorganisms [14–16]. All multicellular organisms live in an intimate and interdependent association with their microbiome, which includes bacteria, archaea, viruses, fungi, and protists [17–20]. Consequently, animals and plants represent functional biological entities comprising a host and its microbiome, so-called metaorganisms [17]. Members of a host-associated microbiota have various functions within a metaorganism and display fundamental roles in host health by contributing, for instance, to host development [21], organ morphogenesis [22], metabolism [23, 24], aging [25], behavior [26], and reproduction [27–29]. Microbes may further be essential for macroorganisms living in extreme environmental conditions [30] and for acclimating and adapting to environmental changes [31–35]. Although the host can also respond to environmental perturbations through phenotypic plasticity [7, 36, 37], microbial-mediated acclimatization has received particular attention. Microbes can play a critical role in controlling host responses to environmental stress through various mechanisms [38], including metabolites and signaling molecules production [39], host stress response stimulation [40], modulation of the host immune response [41], metabolic cooperation [42], biofilm formation [43], and detoxification [44]. Furthermore, microorganisms have shorter generation times, respond more rapidly and are therefore better suited to persist through these stressors [45].

In nature, metaorganisms face a diversity of biotic and abiotic stressors that may require an associated microbial community that responds adequately by changing the composition and/or producing protective molecules or modulating host responses [46]. Consequently, the metaorganism’s fitness may be optimized by altering the composition of its associated microbiota in terms of abundance and/or diversity [30, 34, 45, 47, 48]. Such dynamic restructuring of a host’s community through environmental change is known as microbiome flexibility. For instance, microbiome flexibility has been proposed to play a role in the rapid acclimatization of *Fungia granulosa* after long-term exposure to high-salinity levels [49], acclimatization of *Acropora hyacinthus* to increased thermal stress [32], and the ability of the coral and sponge holobiont to cope with environmental change [34, 50, 51]. [30, 52]However, only a few studies have directly addressed how a microbiome enables acclimatization to short-term changes in a local environment or enables host adaptation (e.g., [53–55, 2019 #4567]). To provide insights into these processes, our research is focused on the microbiome of the moon jellyfish *Aurelia aurita* (Linnaeus, 1758) and its involvement in the eco-physiological responses of that host.

The scyphozoan *A. aurita* is a cosmopolitan species documented worldwide in various coastal and shelf sea environments [56] and is also one of the most frequent blooming jellyfish species [56, 57]. Jellyfish blooms, which are significant and sudden increases in jellyfish populations, have been receiving increased attention in the context of climate change [58]. These blooms can significantly impact marine ecosystems, disrupting the balance of marine food webs and posing threats to biodiversity [59], since jellies predominantly feed on plankton, including fish eggs and larvae. This leads to competition with other planktivorous organisms and potentially reduces food availability for fish and other marine species [60]. The predation pressure from jellies can have cascading effects on the abundance and distribution of various marine organisms, affecting ecosystem stability and biodiversity [61]. With rising sea temperatures and altered ocean currents, climate change is often believed to influence jellyfish population dynamics, facilitating their proliferation. However, the relationship between rising sea temperatures and jellyfish blooms is complex and under scientific discourse. Perceived recent increase in global jellyfish abundance, often seen as a sign of deteriorating oceans, is not conclusively supported by formal analysis of long-term data [62]. While there has been a slight linear increase in jellyfish populations since the 1970s, this trend is not robust and may be part of a larger cyclical pattern. The strongest observed trend indicates that jellyfish populations undergo significant worldwide oscillations with approximately a 20-year periodicity [62]. Nevertheless, the implications of jellyfish blooms extend beyond marine ecosystems, affecting human industries [56]. Commercial fishing, aquaculture, and tourism industries can suffer from jellyfish outbreaks, as these gelatinous creatures can damage fishing gear, clog fishnets, and deter tourists [58]. *A. aurita* blooms are a significant concern in marine ecosystems of the Mediterranean Sea [63], the East Sea [56], the Gulf of Mexico [64], and the Atlantic Ocean [65]. The dynamics of jellyfish blooms, including those of *A. aurita*, are complex and influenced by various factors, including climate change, nutrient inputs, and predator-prey interactions [58]. Understanding the drivers and consequences of these blooms is essential for effective management and conservation strategies. By monitoring jellyfish populations and studying the influencing factors that cause such blooms, scientists and policymakers can develop measures to mitigate the negative impacts of jellyfish blooms and promote the health and resilience of marine environments in the face of climate change [66]. We hypothesize that the microbiome is one of those influencing factors.

**Fig. 1:**
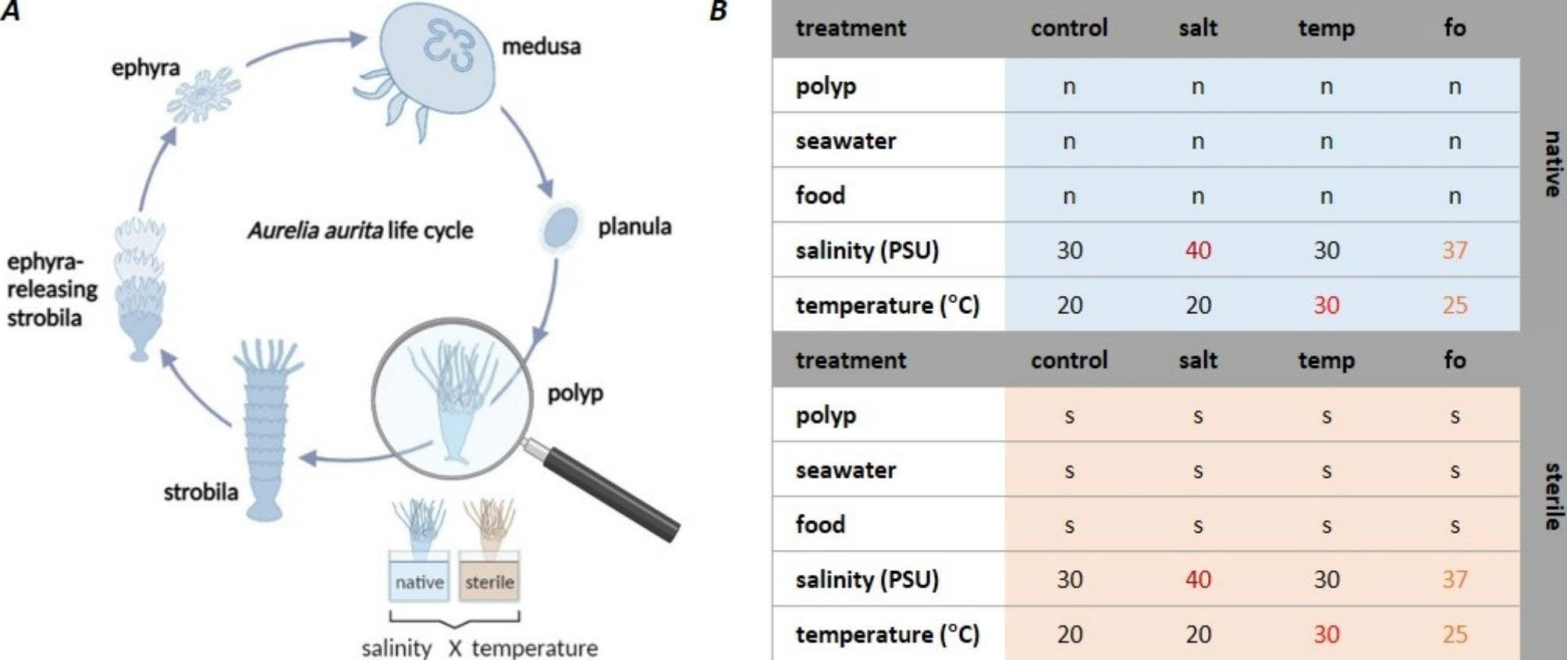
Study design of the host-fitness experiment. (***A***) The life cycle of *Aurelia aurita* alternates between pelagic medusae and benthic polyps. The host-fitness experiments were conducted with polyps exposed to increased temperature and salinity. **(*B*)** Each treatment comprised 96 native (n) or sterile (s) polyps (the latter were kept under sterile conditions throughout the experiment). Control conditions included a salinity of 30 PSU and an ambient temperature of 20 °C. Salinity was increased to 40 PSU (salt) or 37 PSU (fo); temperature was raised to 30 °C (temp) or 25 PSU (fo)

The life cycle of *Aurelia* is biphasic and alternates between free-living pelagic medusae and sessile benthic polyps. While only the medusa can sexually reproduce to form planula larvae, the polyps can undergo asexual reproduction through both budding (clonal polyp generation) and strobilation (production of precursor medusa, i.e., ephyra) (Fig. 1A) [67]. Environmental factors such as temperature, salinity, or food supply influence both the asexual reproduction of the polyps and medusa ecology, such as somatic growth and sexual maturation [68–70]. *A. aurita* is highly flexible and can adapt to a wide range of environmental conditions and survive and reproduce between 4 and 28 °C and 15–38 PSU salinity [71–74]. Temperature plays a crucial role in the reproduction of polyps (e.g., [70, 75–77]. At higher temperatures (20–28 °C), polyps tend to reproduce daughter polyps by budding, while below a certain threshold (< 16 °C), strobilation is triggered to reproduce planktonic ephyrae [70]. Salinity is also expected to determine the settlement of planulae and subsequent development of polyps [56, 78] and may also affect the distribution of polyps in coastal waters (e.g., [75, 79, 80], and the mortality of polyps [80, 81]. Understanding the effect of abiotic factors on the survival and reproduction of *A. aurita* is essential for accurate predictions on the species’ future under climate change and its potential to bloom [56, 82].

The composition and structure of the microbial communities associated with *A. aurita* are well characterized and was shown to be crucial for *A. aurita’s* fitness (survival, feeding, and growth) and particularly for the generation of offspring [29]. Bacterial colonizers belong to various phyla, including Proteobacteria, Firmicutes, Bacteroidetes, and Actinobacteria. Some common bacterial genera in the complex and highly diverse *A. aurita* microbiome include *Vibrio*, *Pseudomonas*, *Pseudoalteromonas*, *Alteromonas*, *Roseobacter*, and *Ruegeria*. The composition of the bacterial communities associated with the moon jellyfish changes with compartment, life stage, and population [29, 83, 84].

Here, we tested how *Aurelia*’s microbiome is changing in composition due to acute temperature and salinity rises, thus affecting host fitness. Ultimately, the microbiome of this metaorganism might mediate the acclimatization of *A. aurita* to climate change, and as a first step in this process, short-term changes were investigated here, which may even help mitigate jellyfish blooms in the future.

## Materials and methods

### *Aurelia aurita* polyp husbandry and generation of sterile polyps

Husbandry and generation of sterile polyps are described in detail in previous studies by Weiland-Bräuer et al. [29, 84, 85]. Briefly, clonally produced polyps of sub-population North Atlantic (Roscoff, France) were kept in the laboratory at 20 °C in 30 PSU artificial seawater (ASW containing 3% w/v Tropical Sea Salts, Tropic Marin) and fed twice a week with freshly hatched *Artemia salina* (HOBBY, Grafschaft-Gelsdorf). These conditions simulate the mean sea surface temperature in summer (20 °C) and salinity (30 PSU) of the North Atlantic Ocean, where these polyps originated [86]. Sterile polyps and brine shrimps *Artemia salina* were generated by treatment of 3-day starved animals with Provasoli’s antibiotic mixture (360,000 U/L penicillin G, 1.5 mg/L chloramphenicol, 1.8 mg/L neomycin, and 9,000 U/L polymyxin B; Carl Roth, Karlsruhe, Germany) in sterile ASW (filtered through 0.22 μm filters). Antibiotic-added sterile ASW was changed daily for three days, and finally, the antibiotics were washed out by water changes for two more days. After five days, the absence of bacteria was confirmed for all putative bacteria-free samples (polyps and brine shrimps) by the lack of amplification of the bacterial 16S rRNA gene with a standard PCR using primer set 27 F and 1492R prior to the experimental start [87]. Sterile polyps were subsequently used for the experiments. Sterile polyps received sterile food in the form of brine shrimps. At the end of the experiment (t_30d_), 24 randomly selected polyps were used for PCR-based sterility check resulting in a lack of amplification, consequently verifying the sterility of polyps over time. Note that “sterile” refers to bacteria-free polyps and brine shrimps, implying that other microorganisms, such as archaea and fungi, remained.

### Challenge of *Aurelia aurita* polyps with environmental stressors

Host fitness experiments were conducted according to Weiland-Bräuer et al., 2020 [29], with a similar setup for native and sterile conditions (Fig. 1). Applied conditions were kept constant throughout the experiments. Single native or sterile polyps were transferred from husbandry tanks to 48-well plates. Each well was filled with 1 mL native or sterile ASW (filtered through 0.22 μm filters), and a single polyp was transferred to the middle of the well. All treatments were simultaneously conducted with 96 replicates each (Fig. 1B). Native and sterile polyps were exposed to control conditions (20 °C and 30 PSU) and high temperatures (30 °C, 30 PSU) or high salinity (40 PSU, 20 °C), without gradual adaptation. Similarly, a future ocean scenario was simulated by combining milder stresses with 37 PSU ASW at 25 °C (Fig. 1B). The latter values were based on a predicted increase of 5 °C and 2 PSU in the year 2500 under the assumption of a 0.1 °C increase per decade and a total salinity rise of 5% [88, 89]. Even at present, heat waves can cause relatively abrupt temperature and salinity anomalies within this range [90–92]. The experimental conditions were maintained for four weeks. During the experiments, the polyps were washed with the appropriate water every two days for the first 14 days of the experiment (monitoring survival rate, growth). Evaporation of the water was not observed, nor was a change in salinity and pH. In week three of the experiment (monitoring feeding rate), the polyps were washed daily after incubation of the food. There was no water change during strobilation and ephyra development. Within the first two weeks and the fourth week, the polyps were fed with freshly hatched (native or sterile) *A. salina* twice a week. There was no washing and feeding during strobilation and ephyrae release.

### Monitoring host-fitness traits

Six different fitness traits: survival, growth, feeding, budding, strobilation, and ephyrae release were monitored. All animals were recorded over time using a stereomicroscope (Novex Binokulares RZB-PL Zoom-Mikroskop 65.500, Novex, Arnhem, the Netherlands) equipped with an HDMI/HD camera. Photos with different exposures, backgrounds, and bright or dark field microscopy were taken to represent the monitored fitness traits adequately. Original photographs presented in this study were processed with Remove.bg. The survival of polyps was assessed every 48 h for the first 14 d based on their phenotypical appearance and the presence of tentacles (Fig. S1A), and accumulative death was calculated at day 14. Growth was documented every 48 h during the same period by measuring the length and width of the polyps (Fig. S1B). Mean start sizes (length multiplied by width at t_0_) and after 14 d (t_14_) were compared per treatment after a gradual change in growth, and growth rates (in %) were calculated. Budding was monitored by counting the number of daughter polyps, and the weekly budding rate was calculated for the first 14 days. The feeding rate of the polyps was monitored for five days during week 3 of the experiments. For this, single polyps were offered 20 *Artemia salina*, and after 1 h, the remaining prey was counted; a mean feeding rate (% of *Artemia* clearance) was then calculated per treatment. Strobilation and ephyrae release were monitored in parallel with a separate set of 96 polyps for each treatment.

For this set of experiments, strobilation was induced by adding 5 µM 5-methoxy-2-methyl indole to the water at days 1, 2 and 3 (involving daily washing and inductor exchange as described in [29]) when exposed to the environmental stress. On day 4, polyps were washed with water to remove the inducer. Immediately after, polyps were monitored for strobilation without being subjected to water changes or feeding,, and strobila phenotypes and the number of segments were detected beginning on day 5 when native control polyps began segmentation. Ephyrae release was monitored each day after their first appearance, and the number of released ephyrae was detected beginning on day 12. Ephyrae release was monitored for the next 4 weeks.

### Data analysis of host-fitness parameters

For each treatment, fitness trait parameters were analyzed, resulting in the following fitness variables: (i) survival, calculated from counts of alive and dead polyps, (ii) % growth rate, (iii) % feeding rate, (iv) % budding rate, (v) counts of segments and the number of ephyrae (strobilation). A log-rank test using the survival library in R (https://www.R-project.org/) was performed to determine the survival rate of polyps [93]. Other fitness variables were assessed using univariate permutational analysis of variance [94]. All fitness variables were tested in the 10 most informative pairwise comparisons between the twelve treatments. PERMANOVAs were performed using R v4.0.0. The vegan package was used for the computations, and the permutation test for adonis was performed under the reduced model with 9,999 permutations [95, 96]. ClustVis web tool (http://biit.cs.ut.ee/clustvis/) was used for visualizing the clustering of multivariate data using heatmap. Clustering distances for rows and columns were calculated with correlation (defined additionally as correlation subtracted from 1). The linkage method included complete linkage.

### 16S rRNA amplicon-based microbiota analysis

16S rRNA amplicon sequencing was performed to analyze the microbial community composition of native polyps. Six native polyps were randomly removed from the 96 replicates before the experimental start and after 14 d. DNA isolation and subsequent 16S rRNA amplicon sequencing were performed as previously described [29]). DNA was isolated using the WIZARD Genomic DNA Purification kit (Promega, Madison, WI, USA), and PCR amplicon libraries of the V1-V2 region of the 16S rRNA gene were constructed using uniquely barcoded primers with primers V1_A_Pyro_27F (5´-CGTATCGCCTCCCTCGCGCCATCAGTCAGAGTTTGATCCTGGCTCAG-3’) and V2_B_Pyro_27F (5’-CTATGCGCCTTGCCAGCCCGCTCAGTCAGAGTTTGATCCTGGCTCAG-3’) combined with 338R. Following amplification in 20 µL, the amplicons were sequenced on an Illumina MiSeq v3 platform (2 × 300 cycle kit) at the Max-Planck Institute for Evolutionary Biology in cooperation with Dr. S. Künzel. 16S rRNA data processing was conducted with mothur v1.39.5 [97] according to the MiSeq SOP (OTUs were detected at a 97% similarity threshold) using SILVA SSU database 138 as described in [29]. Downstream computations for alpha- and beta-diversity analysis were performed in R v4.0.0 using the vegan package (https://www.R-project.org/). R data were imported to Excel for bar plot construction of amplicon data (taxonomic assignment). Sequence data were deposited under the NCBI BioProject PRJNA925707, and BioSample Accessions SAMN32807491- SAMN32807530.

## Results

Host-fitness experiments were conducted with *A. aurita* polyps with a high number of replicates (N = 96) to elucidate the effect of temperature and salinity rises on this host and decipher its microbiota’s role for any acclimatization potential. The host fitness traits of survival, growth, feeding and asexual reproduction were studied under various combinations of increased temperature and salinity (Fig. 1), as these environmental stress conditions are linked to climate change.

### Increased temperature and salinity affect host survival, growth, and feeding rates

**Fig. 2:**
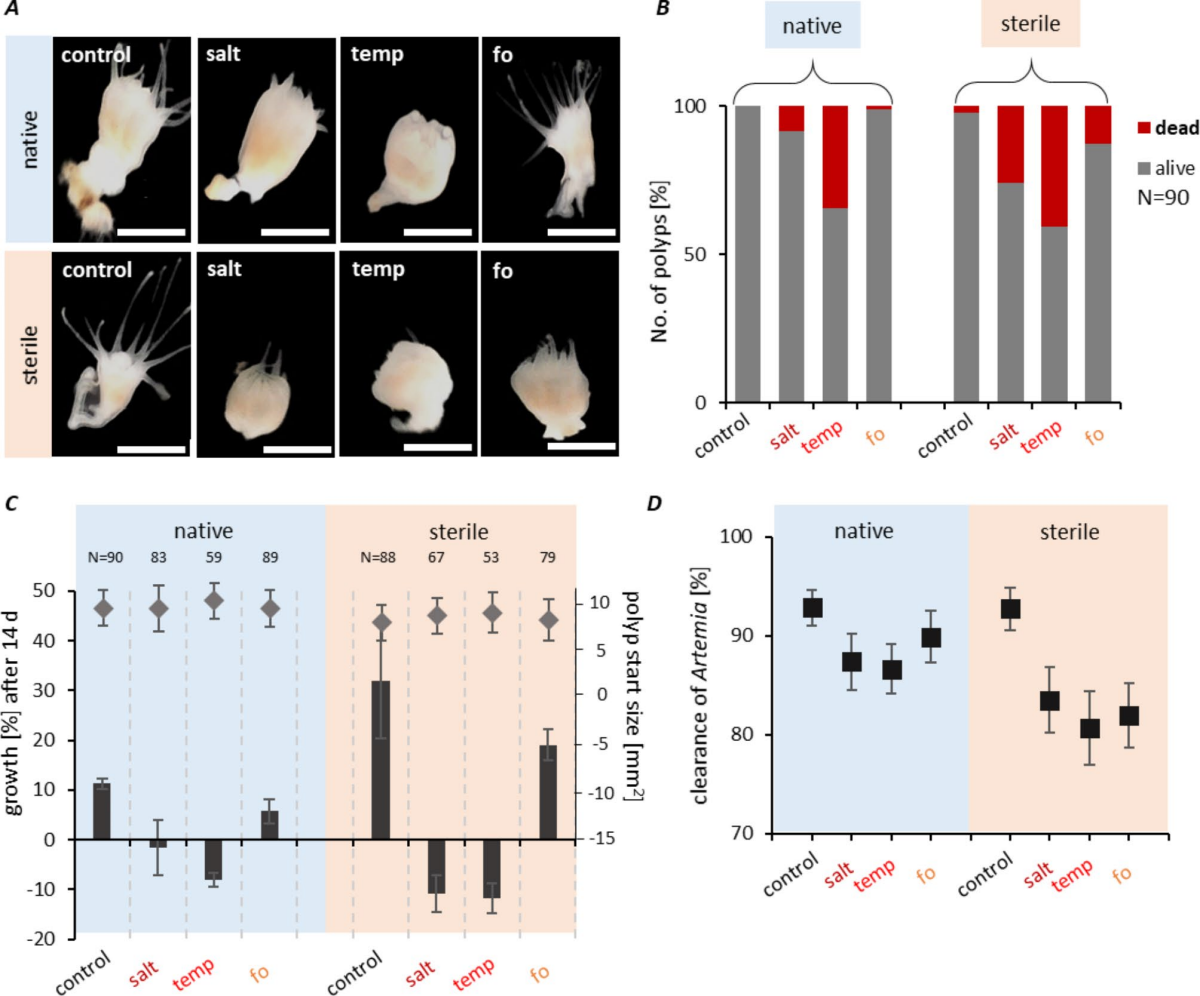
High temperature and salinity impair the fitness of *Aurelia aurita* polyps. (***A*)** Photographs of typical polyps for each treatment after 14 d. Scale bars correspond to 1 mm. **(*B*)** Percentages of dead (dissolved body, no mechanical stimulus triggering) and alive polyps (90 biological replicates) based on polyps’ phenotypical appearance and presence of tentacles. Monitoring was conducted each 48 h, and accumulative numbers are shown after 14 d. **(*C*)** The growth of polyps was followed every 48 h for 14 d by measuring the polyp size (length times width) of live polyps. Mean start sizes (υ, legend at the right) and the corresponding growth rates (%, legend at the left) are plotted, and error bars depict the standard deviations. **(*D*)** Feeding rate (% clearance of 20 *Artemia salina* in 1 h) plotted as mean of single polyps of five monitoring days

The phenotype of native polyps exposed to control conditions, when their survival rate was 100%, is shown in Fig. 2A. All native polyps generally possessed approx. 16 tentacles, and the calyx width increased from 2.67 ± 0.72 mm at the start to 3.56 ± 0.65 mm after 14 d. When the the temperature was increased to 30 °C, polyp survival was significantly reduced (Fig. 2B). The effect of raised salinity on survival was not prominent, giving a 9% reduction (p = 0.250, the outcome of pairwise result of Log-rank test on survival is summarized in Tab. S1). Polyps exposed to high salinity developed a shrunken and widened polyp body (calyx width 4.20 ± 0.74 mm) and absorbance of tentacles (67/90 polyps showed absorbed tentacles) (Fig. 2A, salt treatment). The elevated temperature resulted in a 34% reduction of the survival rate (p-value = 0.003), and live polyps frequently developed an impaired phenotype with absorbed tentacles (73/90 polyps) and a roundish body shape (calyx width 4.26 ± 0.96) (Fig. 2A, temperature treatment). The combination of increased salinity and temperature in a future ocean scenario (fo treatment) produced slight, non-significant effects on polyp survival (99%, p = 0.400; Fig. 2B). Note that the elevation of the single parameters was more extreme (30 vs. 25 °C and 40 vs. 30 PSU) than applied in the combination simulating a future ocean. The observed differences in survival might depend on the extent of the increase in temperature and salinity or on a salt-conveyed thermotolerance.

Similar experiments were also performed with sterile polyps. High-temperature stress lowered their survival rate to 59% (Fig. 2B). Thus, the absence of a microbiome decreased the survival of temperature-stressed animals by a further 6% compared to native polyps (p = 0.600). In the future ocean scenario, the survival rate of sterile polyps was decreased to 87%, which was lower than native animals kept under these conditions (p = 0.200 native vs. sterile in fo, Tab. S1). All stress conditions resulted in impaired phenotypes of live polyps with shrunken and widened body shapes (calyx width in a range of 4.02–4.36 ± 1.05 mm) and absorbed tentacles (65–79 out of 90 replicates). Overall, the survival trends observed with native polyps were exacerbated in sterile animals.

The treated polyps were analyzed for growth after 14 d (Fig. 2C). Under normal conditions, native polyps had a mean size (length multiplied by width) of 9.6 ± 2.08 mm^2^ at the beginning of the experiment, and this gradually increased to 10.6 ± 2.4 mm^2^ after 14 days, corresponding to a growth rate of 11% (Fig. 2C). Exposure to higher salinity halted growth after 14 days (t_0_ = 9.55 mm^2^ to t_end_ = 9.44 mm^2^; Fig. 2C, p < 0.001; the outcome of PERMANOVA tests for growth is summarized in Tab. S2), while high temperatures resulted in downsized polyps (t_0_ = 10.48 mm^2^ to t_end_ = 9.64 mm^2^; Fig. 2C, p-value < 0.001). Under future ocean conditions, the polyps could grow (t_0_ = 9.59 mm^2^ to t_end_ = 10.14 mm^2^), albeit significantly less than under control conditions (growth rate 6%, Fig. 2C, p < 0.001). Sterile animals were initially slightly smaller than native animals (t_0_ = 8.00 mm^2^), but during growth, generally, even higher growth rates than observed for native animals were achieved (t_end_ = 10.56 mm^2^; growth rate 32%; Fig. 2C). Growth did not occur in the absence of microbes under high salinity (t_0_ = 8.75 mm^2^ to t_end_ = 7.80 mm^2^) or high temperature (t_0_ = 9.12 mm^2^ to t_end_ = 8.04 mm^2^). The shrinking effect under salt stress was substantial, where sterile polyps decreased their size by 9.3% compared to halted growth of native animals (Fig. 2C, p < 0.001). Furthermore, the sterile animals shrank more strongly at high temperatures than the native polyps (-11.8% vs. -8.0%, p = 0.012).

Feeding rates of live polyps were assessed during week three of the experiments for five consecutive days (Fig. 2D). Native polyps kept under control conditions had a mean *Artemia* clearance rate of 92.8% ± 8.9% (Fig. 2D). High salinity or high temperature caused a reduction to 87.4% and 86.6%, respectively (Fig. 2D, p = 0.03 and p = 0.036; see Tab. S3 for PERMANOVA tests on feeding). The feeding rate of sterile polyps was significantly reduced (p < 0.02) under all stress conditions compared to the control treatment of those animals (Fig. 2D). There was no statistical difference in feeding rates between native and sterile animals kept under the same conditions (Tab. S3). Thus, the increased growth of sterile animals compared to native polyps under the same condition was not due to increased feeding.

### Increased salinity and temperature affect the asexual reproduction of the host

**Fig. 3:**
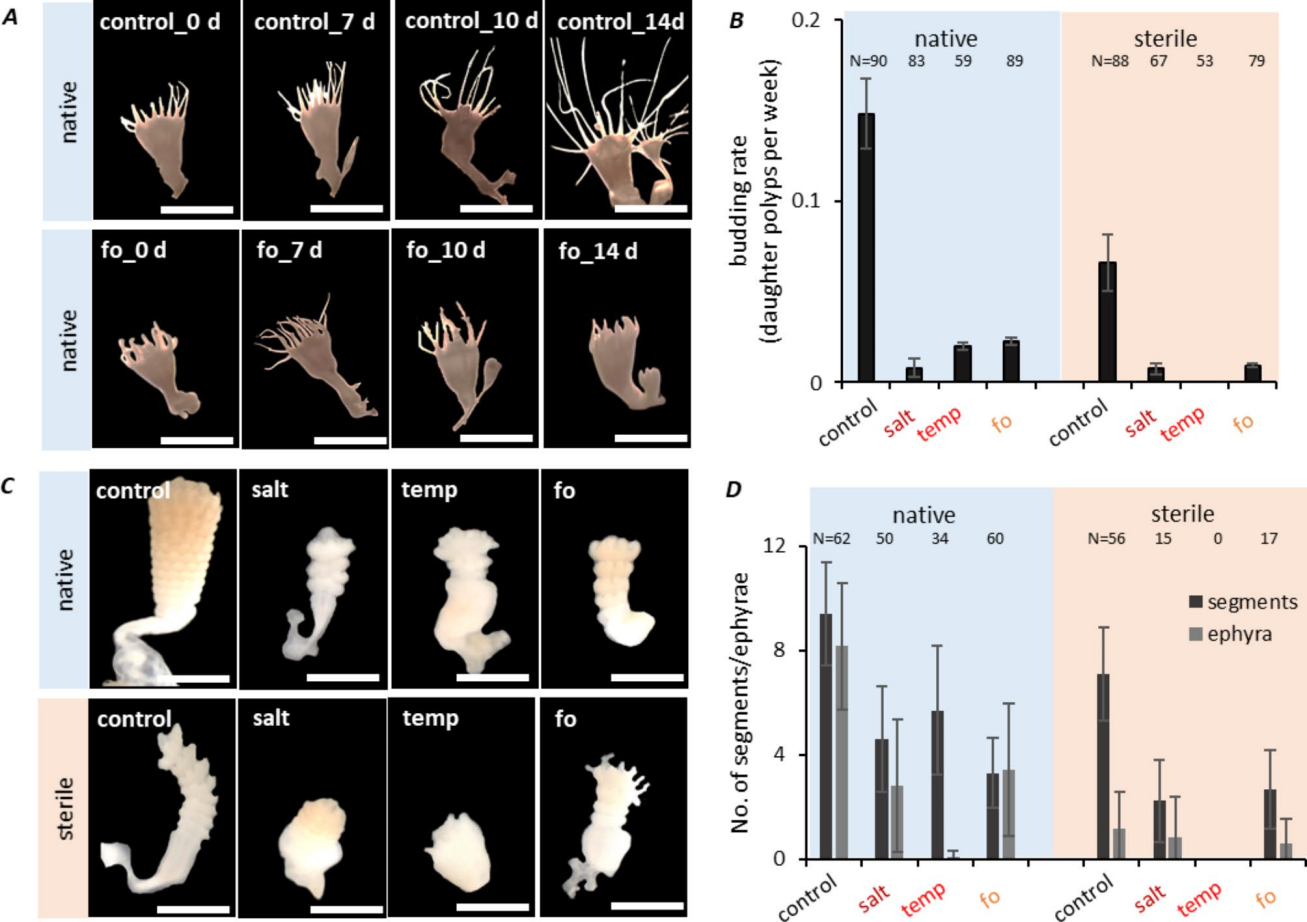
Asexual reproduction of *Aurelia aurita* polyps diminished under ambient stress conditions. (***A*)** Daughter polyp generation of native polyps under control and future ocean conditions followed for 14 d. Scale bars correspond to 2 mm. **(*B*)** Budding was followed every 48 h for 14 d by monitoring the generation of daughter polyps and calculated as daughter polyp generation per week. Error bars depict the standard deviations from the calculated mean. **(*C*)** Strobilation of polyps was induced using 5 µM 5-methoxy-2-methyl indole. Photographs show the segmentation of polyps for native and sterile polyps under control conditions six days post-induction. Scale bars correspond to 2 mm. **(*D*)** Formation of strobilae was monitored daily and quantified by the number of segments formed per strobila. The numbers of released ephyrae were determined for 4 weeks. Error bars depict the standard deviations from the calculated mean

To determine the effect of the environmental stressors on the asexual reproduction of *A. aurita*, the generation of daughter polyps was monitored every 48 h over 14 days (Fig. 3A). Under control conditions, native polyps showed an average budding rate of 0.15 daughter polyps per week (Fig. 3B). They generated up to 2 daughter polyps per week (9% produced one daughter polyp, and 3% resulted in two daughter polyps). Budding of native polyps was significantly negatively affected in all stress treatments (Fig. 3A, B; p < 0.001, see Tab. S4 for PERMANOVA tests on budding). Under all stress conditions, fewer polyps were produced that lacked tentacles (example photographs are shown for the fo condition in Fig. 3A). In the absence of bacteria, budding was seriously impaired. Under control conditions, the budding of sterile polyps was decreased by 45% (Fig. 3B, p < 0.001, Tab. S4), in line with previous observations [29]. Only 8% of the sterile animals produced one daughter polyp, and none produced two. The applied environmental stress conditions lowered the reproduction rates even further (Fig. 3D, p < 0.001 for all sterile conditions compared to sterile control treatment, Tab. S4); no daughters were produced at all by sterile polyps under high temperature (Fig. 3A, B).

Following chemical induction, native polyps kept under control conditions began strobilation by initiating the segmentation of the prolonged body (early strobila) on day 5. Here, segmentation was completed in the late strobila stage on day 9 (Fig. 3C), and by then, 62 of the 96 replicate native polyps (65%) had formed a strobila (indicated by the number of strobilae above Fig. 3D). Raising the salinity and temperature significantly reduced the formation of strobilae, which were now only visible in 50 and 34 polyps, respectively (52% and 35%, a significant reduction compared to control p < 0.001, Tab. S5). The number of segments formed per polyp under the various treatments on day 9 is shown in Fig. 3D. The number of segments of a strobila reflects the reproductive output of a polyp. However, due to stress, these segments may not be formed correctly and/or may not be released as ephyra. The difference between the number of segments and ephyra release thus indicates a stress-induced disturbance of ontogenesis [29]. All stress conditions of native polyps resulted in fewer numbers of segments (p < 0.001, Tab. S5), and subsequently, fewer ephyrae were released by native animals under stress conditions (Fig. 3D, p < 0.001, Tab. S6). The formation of strobilae was not only impaired but also delayed, and abnormal phenotypes were formed (Fig. 3C), including malformed structures, incomplete or completely missing constriction, impaired tentacle absorption, and colorless, minimized, and partly widened bodies (Fig. 3C). Compared to control conditions, in which native polyps constricted a mean of 9.4 segments to release eight ephyrae (Fig. 3D), environmentally stressed polyps formed only three to five segments and released between zero and 3.4 ephyrae (Fig. 3D). The most potent effect was observed at high temperature (6 segments, no ephyrae, Fig. 3D; p < 0.001). The elevated temperature and salinity levels, alone or in combination as in the future ocean scenario, produced even more substantial negative effects on asexual reproduction without the microbiota. Crucially malformed strobilae were monitored, showing only slight constrictions, which went hand in hand with massively reduced ephyrae release (0 to 0.8; p < 0.001). The offspring’s generation was halted entirely at raised temperatures without a microbiota (Fig. 3C, D).

### Environmental stress conditions cause changes in microbial community patterns that correlate with reduced host fitness

**Fig. 4:**
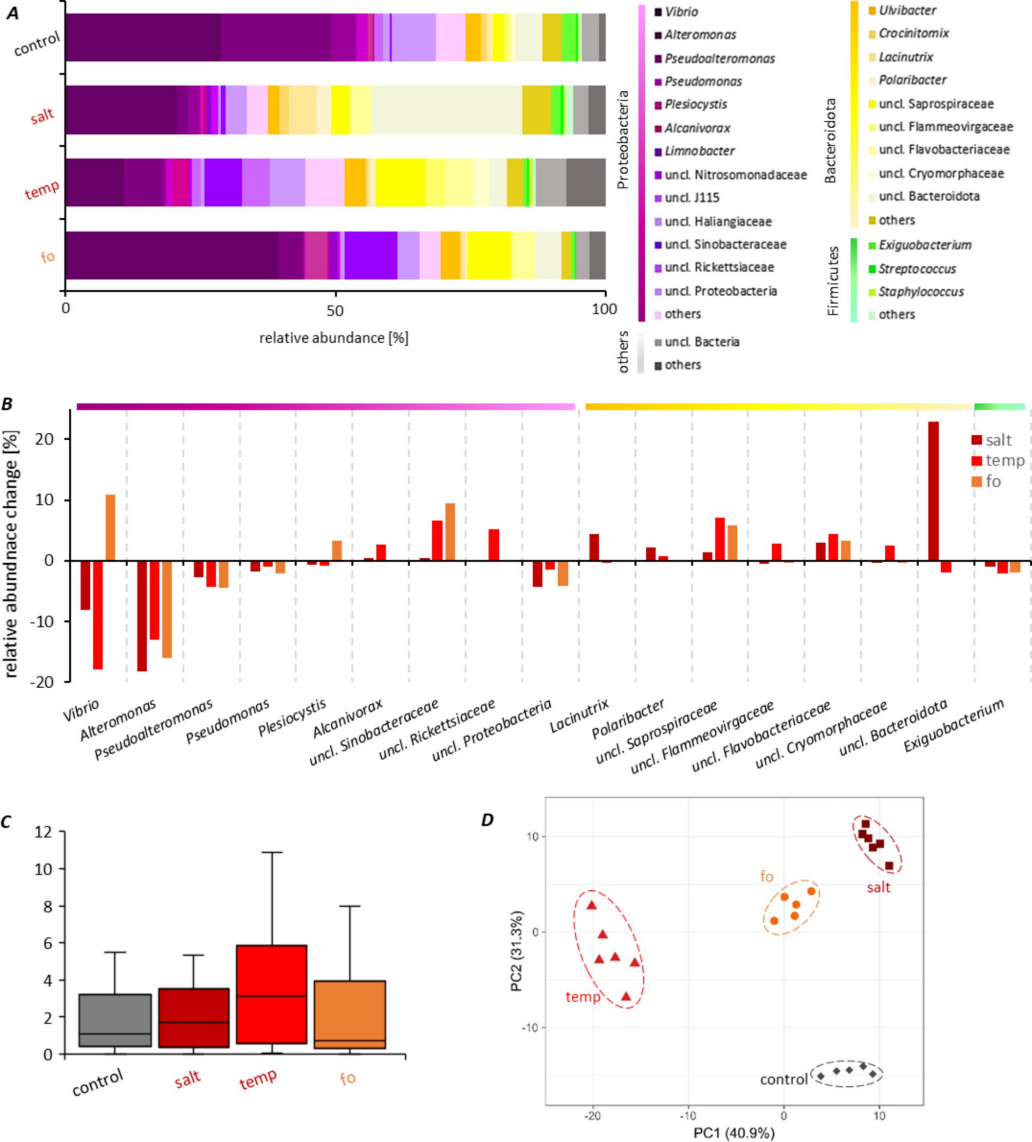
Microbial community composition of *Aurelia aurita* polyps under environmental challenge. Microbial communities were analyzed by sequencing the V1-V2 region of the bacterial 16S rRNA gene. OTU abundances were summarized at the genus level and normalized by the total number of reads per sample. **(*A*)** Bar plots visualizing the dominant genera (reaching at least 1% of relative abundance) after 14 days of maintenance under control and at the indicated conditions. All data are based on the means of 6 biological replicates. **(*B*)** Differences in relative abundances at day 14 compared to control conditions for all dominant genera. **(*C*)** Boxplot of alpha-diversity Shannon indices. **(*D*)** Principal component analysis (PCA) for genus-level microbiota of the polyps (replicates of the same treatment are grouped with polygons). Different colors and shapes denote treatments and ellipses representing 95% confidence interval for the centroids of each data cluster

Six native polyps were randomly taken from the 96 replicates for each treatment before the experimental start and after 14 d, and 16S rDNA V1-V2 amplicon sequencing was performed to characterize the polyp microbiota. A subset of 2,300 sequences per sample was generated to eliminate bias due to unequal sampling. In total, 946 OTUs were identified, and of these, 461 OTUs were shared by all biological replicates of all treatments. Microbial composition patterns showed no significant change over time (p > 0.2) when comparing t_0_ and t_14d_ samples. Therefore, t_14d_ was used as control for comparing treatment effects. Phylogenetic analysis of the samples revealed a complex microbiota structure that changed due to exposure of the polyps to environmental stressors (Fig. 4). The reproducibility between the six replicates per treatment was high, and thus, the means of the replicates were reported to better resolve differences caused by stressful conditions. At the phylum level, the microbiome of polyps kept under control conditions was composed of 74% Proteobacteria and 18% Bacteroidota, with 4% reads derived from Firmicutes and 3% unclassified (uncl.) bacteria, whereas bacterial lineages with < 1% relative abundance (collectively reported as “others”) accounted for 1% (Fig. 4A). Within the phylum Proteobacteria, *Vibrio* and *Alteromonas* accounted for the largest proportion; Bacteroidota were mainly represented by *Ulvibacter* and uncl. Bacteroidota. Although Proteobacteria, Bacteroidota, Firmicutes, and uncl. Bacteria remained the most abundant phyla also under stress conditions, shifts were observed (Fig. 4A). The higher salinity resulted in a stark reduction in Proteobacteria (now only comprising 38%) in favor of Bacteroidota (now 53%), while Firmicutes and uncl. Bacteria remained almost constant. Although not quite as intense, similar shifts were observed at high temperatures. Here, 52% Proteobacteria and 34% Bacteroidota were assigned, while Firmicutes were detected with 2% and uncl. Bacteria with 6% (Fig. 4A). In comparison, the future ocean treatment showed weaker changes on phylum level compared to control conditions, giving 70% Proteobacteria, 24% Bacteroidota, 1% Firmicutes, 2% uncl. Bacteria, and 3% others (Fig. 4A). Major shifts at the genus level are summarized in Fig. 4B. For all stress treatments, an increase of at least 1% was observed for uncl. Sinobacteraceae, uncl. Saprospiraceae and uncl. Flavobacteriaceae, at the expense of *Alteromonas, Pseudoalteromonas, Pseudomonas*, uncl. Proteobacteria and *Exiguobacterium* (Fig. 4B).

*Vibrio* showed a decline in salt and temperature treatments, whereas an increase was recorded for the future ocean scenario (Fig. 4B). *Alcanivorax*, uncl. Rickettsiaceae, uncl. Flammeovirgaceae, and uncl. Cryomorphaceae increased in relative abundance exclusively after temperature increase (Fig. 4B), while *Lacinutrix* and, in particular, uncl. Bacteroidota proliferated under salt stress (Fig. 4B). Despite these differences, the alpha-diversity (species richness and evenness as calculated by the Shannon index) of the polyps’ microbiota did not significantly differ between treatments. However, the range between replicates was notably smaller in the salt treatment compared to the two other treatments (Fig. 4C). Beta-diversity was assessed by Principal component analysis (PCA) at the genus level to summarize the marked differences in community composition of the individual polyps (Fig. 4D). The first two axes of the generated PCA plot explain 72.2% of the variation of the analyzed communities, that were separated into four clusters corresponding to the treatments. The microbiota resulting from high temperature produced the highest variance in bacterial composition compared to control conditions. High salinity and the mild but combined heat- and saline stress of the future ocean had less impact on the compositional variance, suggesting that the variance between the bacterial compositions depends on the strength and the applied environmental stress, whereby the effects differ for each applied condition (Fig. 4D).

**Fig. 5:**
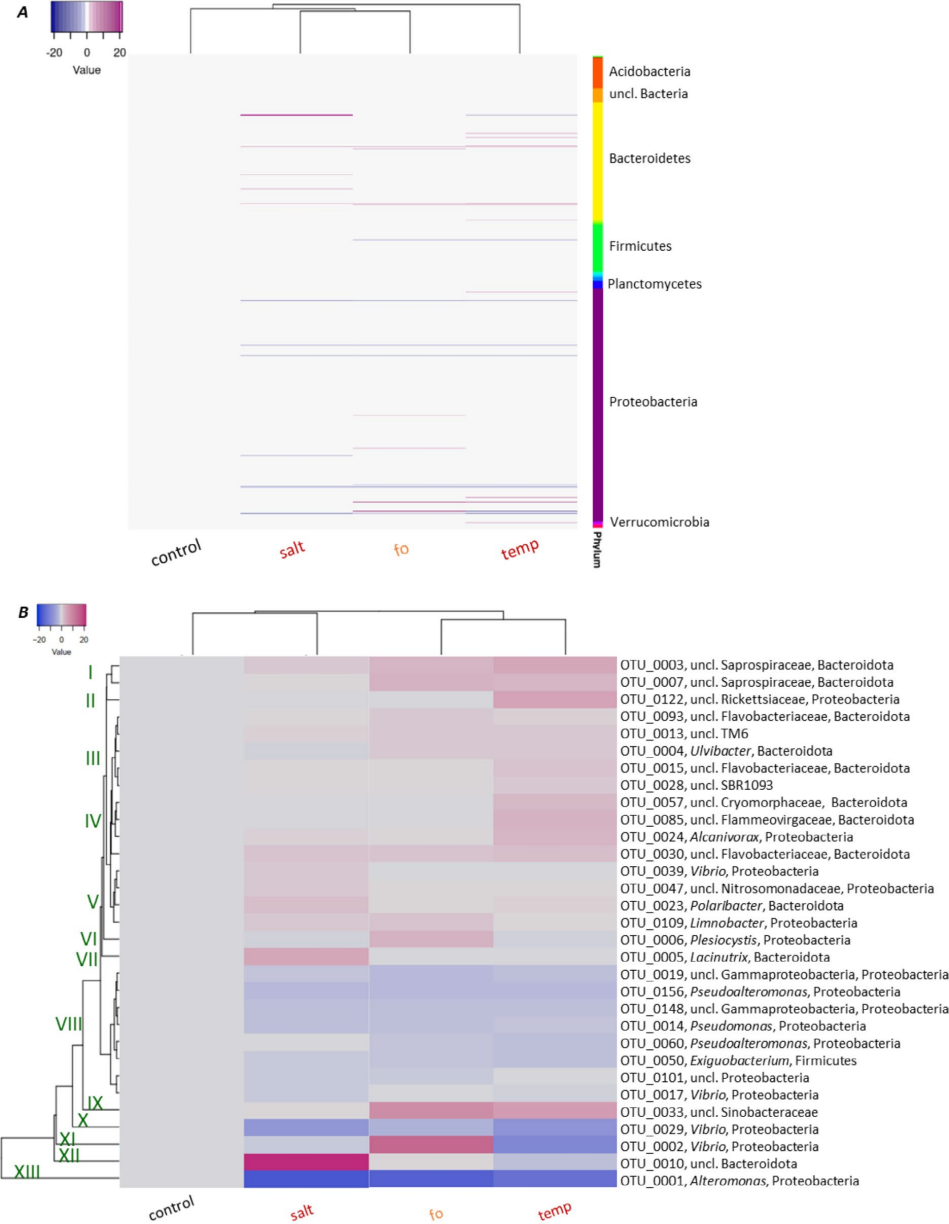
In-depth microbial community analyses. Heatmap and hierarchical clustering (based on the complete correlation of conditions) of OTUs in the microbiomes of polyps after 14 days of environmental challenge. Each column represents the mean of 6 replicates per treatment. Each row represents an OTU. The colors indicate fold changes of relative abundance compared to control conditions (blue, decreased abundance; red, increased abundance). **(*A*)** Holistic analysis of the identified 461 OTUs assigned to their phylum. **(*B*)** Heatmap and hierarchical clustering (cluster I – XIII) of most abundant OTUs (> 1% relative abundance in control conditions) in the microbiomes

Hierarchical cluster analysis verified the different community structure profiles obtained from all conditions as visualized on genus level in Fig. 4 by resolving the observed microbiota dynamics on OTU level. An initial analysis considered all shared 461 OTUs and identified their changes in relative abundances compared to the control treatment. These were grouped taxonomically in the heatmap, visualizing their fold-change (Fig. 5A). Shifts can be seen across the whole microbial community within the abundant phyla; however, 26% of the OTUs (122 of 461) remained constant (cutoff 0.2% relative abundance change, p < 0.0001) compared to the control conditions. The combination of salinity and temperature stress in the future ocean scenario not only resulted in specific changes but also reflected the effects of separate elevated temperatures or salinity. We next zoomed in on the 31 most abundant OTUs (relative abundance > 1% in the control), as their shifts explained the major differences in the community composition after the applied stresses (Fig. 5B). Hierarchical clustering of those OTUs allowed the identification of thirteen (I-XIII) clusters showing similarities in relative abundance changes compared to the control. By far, the strongest effects were seen for OTU 001 (*Alteromonas*, strongly decreasing in all three treatments) and OTU 0010, an unclassified Bacteroidota that strongly increased with high salt. Eight OTUs decreased mildly in abundance to all conditions (OTUs 0019, 0156, 0148, 0014, 0060, 0050, 0101, 0017 in cluster VIII) and OTUs 0029 and 0001 decreased more strongly, while 21 OTUs increased to most of the stressors (Fig. 5B). However, only OTU 0122 (an uncl. Rickettsiaceae member of the Proteobacteria) remained constant under salt and fo conditions compared to the control. Of the increased OTUs, twelve proliferated under all stress conditions (OTUs 0003, 0007, 0093, 0013, 0015, 0028, 0024, 0030, 0047, 0023, 0109, and 0033). An increase due to future ocean conditions but a decrease in relative abundance through individual salt and temperature treatment was detected for OTUs 0002 and 0006. OTUs 0039 and 0005 were raised due to salt stress but declined under temperature and fo conditions. Two OTUs (0085 and 0057) increased with temperature while decreasing under salt and fo treatments. Only OTU 0004 increased under temperature and fo conditions, while OTU 0010 proliferated under salt and fo conditions (Fig. 5B).

**Fig. 6:**
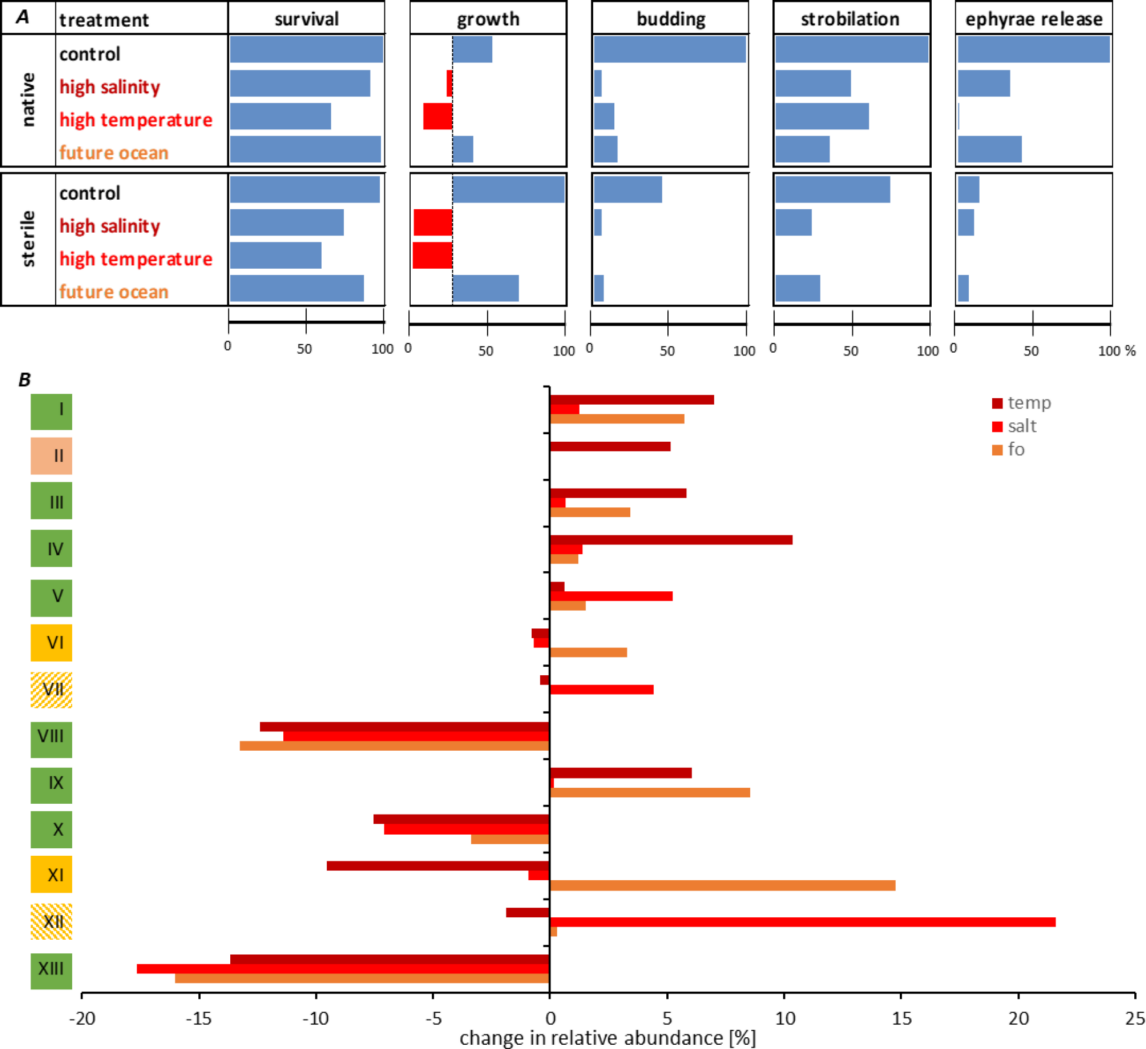
Impact of environmental changes on *Aurelia aurita* fitness traits in correlation to microbial community changes. (***A***) Fitness parameters. All data are expressed as % increase or decrease relative to control native animals taken as 100%. Feeding rates are not shown as they did not significantly vary. (***B***) Relative abundance changes (%) of highly abundant, defined OTU clusters I-XIII (see Fig. 5B) through environmental stress compared to native normal conditions

Lastly, we combined the observations on fitness effects and changes in microbial community patterns through environmental stressors graphically in Fig. 6. The fitness data illustrate that, first, high temperature and high salinity, in that order, have the most significant adverse effects on polyp fitness (Fig. 6A). It is further demonstrated that sterile animals are even more affected. Second, growth and asexual reproduction are the fitness traits most severely affected (Fig. 6A). Impaired fitness correlated with the absence of the microbiota and its compositional change (Fig. 5). In Fig. 6B, it can be seen that clusters I, III, IV, V, VIII, IX, X, and XIII showed the same trend in compositional change regardless of the environmental stress compared to normal native conditions. Bacterial OTU clusters I, III, IV, V, and IX increased in relative abundance compared to normal conditions (highlighted in green in Fig. 6B), while OTU clusters VIII, X, and XIII declined (highlighted in green in Fig. 6B). Thus, loss of fitness correlated with the decrease of representatives from the genera *Alteromonas*, *Exiguobacterium Pseudoalteromonas*, *Pseudomonas*, and *Vibrio* of Proteobacteria, and the increase of Bacteroidota represented by *Polaribacter*, *Ulvibacter*, uncl. Flavobacteriaceae, uncl. Flammeovirgaceae, uncl. Sinobacteraceae, and uncl. Saprobacteraceae. OTU clusters VI (*Plesiocystis*) and XI (*Vibrio*) (highlighted in yellow in Fig. 6B) showed a decline under single stress, but a rise in the future ocean scenario. The clusters VII (*Lacinutrix*) and XII (uncl. Bacteroidota) (yellow striped in Fig. 6B)indicated a decrease (VII) or increase (XII) under stress conditions based on 2 of the three tested stress conditions (they showed the inverted effect in the third condition). The increase of cluster II (uncl. Rickettsiaceae) was exclusively observed for high-temperature conditions. In summary, high temperature and salinity have the most significant adverse effects on polyp fitness, especially when the animals are sterile. However, the change in bacterial community patterns, expressed by the decrease of representatives from Proteobacteria and the increase of OTUs of Bacteroidota, was also associated with the loss of fitness.

## Discussion

Over the past 100 years, the sea surface temperature has increased on average by 0.6 °C [98]. Moreover, more frequent climatic extremes, like marine heatwaves, result in animal performance declines, mitigation, and local mortality [99–101]. In addition to temperature changes, historical records show that the ocean salinity increased by 4% between 1950 and 2000 [102]. Many marine species are stenohaline and cannot tolerate a wide fluctuation in the salinity of water; thus, their narrow range of salt tolerance limits their survival, reproduction, and germination [103]. Salinity can act synergistically or antagonistically with other environmental stressors; for instance, salt stress was reported to cross-protect thermal stress [104–106]. The capability of marine animals to adapt to future ocean scenarios is crucial for maintaining biodiversity and ecosystem functions [35]. Host-associated microbial communities represent a major factor regulating the host’s response to their external environment [31–35]. Change in the composition of a host’s microbiome (both loss of taxa, shifts in relative abundance, or appearance of novel taxa) has been linked to adapted host fitness as a function of environmental change [34, 107]. Correlative observational studies were reported for salt stress in algae [108], thermal tolerance in sea anemones [109, 110], and heat stress in corals and sponges [32, 111–113]. Consequently, a shift in the microbiome toward a microbial community that supports host fitness could reinforce rapid host acclimatization [114]. High microbiome flexibility may promotes metaorganismal acclimatization, at the risk of losing putatively essential associates and possibly allowing pathogen invasion [34]. Low microbiome flexibility in *Pocillopora* coral was linked to coral disease outbreaks, whereas high microbiome flexibility in *Acropora* corals was linked to rapid adaptation to escape the disease [51].

The microbiome of *A. aurita* benefits the host in adjusting to changes in the environment, such as temperature and salinity, and plays a supportive role for host acclimatization. A diverse and flexible microbiome might assist in maintaining host fitness in a climate-changed ocean. This assumption is supported by our host-fitness experiments conducted with sterile animals, which led to losses in survival, growth, and progeny output under standard (though sterile) conditions, exacerbated under environmental stressors (Figs. 2 and 3). We had already demonstrated in a previous study that bacteria function as a protective shield, and their absence impaired host fitness and affected life cycle decisions, resulting in the halt of offspring generation [29]. These results were verified with sterile polyps under standard conditions, which were also almost completely impaired in asexual reproduction, especially in the release of the ephyrae. Here, we additionally demonstrate that the associated microbiota of *A. aurita* is changing in composition due to acute, sublethal temperature and salinity increases. This consequently affects host survival, and for those polyps that do survive, growth and asexual reproduction are impaired (Fig. 2). Note that energy-intensive fitness parameters related to reproduction were more affected than mere survival. Raising the salinity and temperature to sublethal levels impaired all analyzed fitness traits, leading to 66% reduced survival rates and halting offspring generation. Several studies observed that environmental stressors diminish invertebrate reproduction (i.a., [115, 116]); however, only a few studies link microbiome shifts of these hosts to those effects [117].

We assume that changes in the microbial composition support acclimatization by the host, but drastic changes are associated with loss of microbial function, causing fitness deficits. In a natural setting, exposure to changed salinity or temperature may be more gradual than the abrupt changes applied here, possibly allowing for a slow but steady adaptation of the microbiome and its host. Nevertheless, during heatwaves [99–101], which are expected to increase in severity and frequency due to climate changes, local temperature and salinity changes can be relatively rapid, especially in shallow waters [99–101]. When more moderate increases of salinity and temperature were combined in a future ocean scenario, this resulted in a less impaired fitness than for the more severe, single stressors (Fig. 6A). This showed that the effects on host fitness correlate with the strength of the environmental stress, while salt-conveyed thermotolerance may also be involved. To our knowledge, salinity-conveyed thermotolerance in marine macroorganisms has only been described in corals [23, 118] and data on *A. aurita* are lacking. Currently, it is unknown whether the bacterial community patterns and the response of the corals to different salinities are causally linked or whether they represent parallel responses of the host and its associated bacteria [110]. Recent studies propose that osmolytes like floridoside might play a role in adjusting osmotic pressure by counteracting oxidative stress due to combined salinity and heat stress, thereby contributing to stress resilience [23, 118]. Similar studies would need to be conducted with *A. aurita* to gain deeper insights into the salinity-driven thermotolerance of this host.

Following analysis of the polyp’s microbiomes, we observed major changes in relative abundance that occurred on phylum, genus, and OTU levels (Figs. 4, 5 and 6). Highly-abundant genera like *Alteromonas*, *Pseudoalteromonas*, and *Pseudomonas* (all Proteobacteria) declined under all environmental stress conditions, while various unclassified genera assigned to Proteobacteria and Bacteroidota increased. Notably, some *Vibrio* OTUs increased, whereas others decreased (depending on the condition), indicating that reporting findings on genus level only can be imprecise. We demonstrate that approximately a quarter of the detected community members (26%) maintain their relative abundance irrespective of environmental change. Other members may be interchangeable and act as microbiome regulators that maintain a constant microbiome functionality, irrespective of individual members during environmental change. Alternatively, those bacterial members that change in abundance due to environmental conditions may represent microbiome conformers that adapt to their surrounding environment and change the functionality of the complete microbiome [51]. We noted that the 31 most abundant OTUs all changed their abundance as a result of environmental stress (Fig. 5B), and the intensity of the environmental stressor drives the degree of community change. Thermal tolerance of animals is assumed to be associated with an increase in Alpha- and Gamma-Proteobacteria [35, 119, 120]. That was not observed in our experiments, as the Proteobacteria phylum decreased under high salt or high temperature conditions. Alpha-Proteobacteria produce protecting antioxidants within the coral holobiont [121], and Gamma-Proteobacteria representatives inhibited the growth of coral pathogens and provided additional nutrients for the host [122, 123]. Clearly, such observations cannot be generalized and extended to different hosts, such as jellyfish. Impaired fitness of *A. aurita* polyps correlates with complex abundance shifts on the OTU level. Our results suggest that microbial communities play a critical role in affecting the response of animals to ambient temperature and salinity. Recent studies have suggested that the microbiome might be crucial in mediating the resilience of marine organisms, including jellyfish, to climate change stressors [58, 124]. A healthy and diverse microbiome could enhance the host’s ability to withstand environmental challenges and promote overall ecosystem stability [125, 126]. Consequently, the metaorganism concept should be considered for predicting species’ responses to global climate change. Climate change producing warmer ocean temperatures and increased salinity may enhance jellyfish reproduction and growth rates, leading to population booms. This study’s simulated future ocean scenario demonstrated that jellyfish bloom-causing *A. aurita* can adapt and survive under changing environmental conditions. The relationship between the host’s microbiome, stress tolerance, and climate change concerning jellyfish blooms is complex and likely involves numerous interacting factors. Understanding these intricate connections is essential for predicting and managing jellyfish blooms in the context of ongoing climate change. By gaining a deeper understanding of these processes, thus implementing the metaorganism concept, researchers can develop more effective strategies for managing and mitigating the impacts of jellyfish blooms in the context of a changing climate.

## Conclusions

The role of metaorganism’s microbiomes in host fitness and ecological interactions is increasingly evident. *A. aurita* is one of the main contributors to jellyfish blooms that cause enormous ecological and socioeconomic damage, and this study identifies the response of its microbiome to environmental challenges, coinciding with changes in the fitness of the polyps. A microbiome’s presence is beneficial for these animals’ stress tolerance, and microbial community changes correlate with impaired host fitness of *A. aurita* when the temperature or salinity is increased to sub-lethal levels. In a future ocean scenario, mimicked here by a combined but milder rise of temperature and salinity, the fitness of polyps was less severely impaired, together with condition-specific changes in the microbiome composition. Our results show that the effects on host fitness correlate with the strength of environmental stress, while salt-conveyed thermotolerance might be involved. Microbiome-mediated acclimatization and adaptation may provide a mechanism for hosts besides phenotypic plasticity. Thus, microbiome flexibility can be a fundamental strategy for marine animals to adapt to future ocean scenarios to maintain biodiversity and ecosystem functioning.

### Electronic supplementary material

Below is the link to the electronic supplementary material.

Supplementary Material 1. Tables S1-S6: PERMANOVA tests.: S1-Survival, S2-Growth, S3-Feeding rates, S4-Budding, S5-Segmentation (strobilation), S6-Ephyrae release. Figure S1: Monitoring polyp fitness traits survival and growth.

## Data Availability

All data supporting the findings of this study are available within the paper and its Supplementary Information. Beyond, sequence data were deposited under the NCBI BioProject PRJNA925707, and BioSample Accessions SAMN32807491- SAMN32807530.

## References

[1] Consortium GMR. World Ocean Review 7 - living with the oceans. In.; 2021.

[2] Buck-Wiese H, Voolstra CR, Brüwer J. The metaorganism frontier–incorporating microbes into the organism’s response to environmental change. In: YOUMARES 7 conference proceedings. 2016: 94–102.

[3] Leal Filho W, Nagy GJ, Martinho F, Saroar M, Erache MG, Primo AL (2022). Influences of Climate Change and Variability on Estuarine Ecosystems: an impact study in selected European, South American and Asian Countries. Int J Environ Res Public Health.

[4] Ducklow H, Cimino M, Dunton KH, Fraser WR, Hopcroft RR, Ji R (2022). Marine pelagic ecosystem responses to climate variability and change. Bioscience.

[5] Fuhrman JA (2009). Microbial community structure and its functional implications. Nature.

[6] Reusch TBH (2014). Climate change in the oceans: evolutionary versus phenotypically plastic responses of marine animals and plants. Evol Appl.

[7] Foo S, Byrne M (2016). Acclimatization and adaptive capacity of marine species in a changing ocean. Adv Mar Biol.

[8] Abirami B, Radhakrishnan M, Kumaran S, Wilson A (2021). Impacts of global warming on marine microbial communities. Sci Total Environ.

[9] Sutherland WJ, Freckleton RP, Godfray HCJ, Beissinger SR, Benton T, Cameron DD (2013). Identification of 100 fundamental ecological questions. J Ecol.

[10] Martini S, Larras F, Boyé A, Faure E, Aberle N, Archambault P (2021). Functional trait-based approaches as a common framework for aquatic ecologists. Limnol Oceanogr.

[11] Vergés A, Doropoulos C, Malcolm HA, Skye M, Garcia-Pizá M, Marzinelli EM et al. Long-term empirical evidence of ocean warming leading to tropicalization of fish communities, increased herbivory, and loss of kelp. Proceedings of the National Academy of Sciences. 2016;113(48):13791-6. 10.1073/pnas.1610725113 PMC5137712 27849585

[12] Provost EJ, Kelaher BP, Dworjanyn SA, Russell BD, Connell SD, Ghedini G (2017). Climate-driven disparities among ecological interactions threaten kelp forest persistence. Glob Change Biol.

[13] Qiu Z, Coleman MA, Provost E, Campbell AH, Kelaher BP, Dalton SJ (2019). Future climate change is predicted to affect the microbiome and condition of habitat-forming kelp. Proc Royal Soc B.

[14] Harvell CD, Mitchell CE, Ward JR, Altizer S, Dobson AP, Ostfeld RS (2002). Climate warming and disease risks for terrestrial and marine biota. Science.

[15] Hutchins DA, Fu F (2017). Microorganisms and ocean global change. Nat Microbiol.

[16] Hutchins DA, Jansson JK, Remais JV, Rich VI, Singh BK, Trivedi P (2019). Climate change microbiology—problems and perspectives. Nat Rev Microbiol.

[17] Bosch TC, McFall-Ngai MJ (2011). Metaorganisms as the new frontier. Zoology.

[18] Faure D, Simon J-C, Heulin T (2018). Holobiont: a conceptual framework to explore the eco-evolutionary and functional implications of host–microbiota interactions in all ecosystems. New Phytol.

[19] Jaspers C, Fraune S, Arnold AE, Miller DJ, Bosch T, Voolstra CR. Resolving structure and function of metaorganisms through a holistic framework combining reductionist and integrative approaches. Zoology. 2019. 10.1016/j.zool.2019.02.007 30979392

[20] Hentschel U (2021). Harnessing the power of host–microbe symbioses to address grand challenges. Nat Rev Microbiol.

[21] Rook G, Bäckhed F, Levin BR, McFall-Ngai MJ, McLean AR (2017). Evolution, human-microbe interactions, and life history plasticity. Lancet.

[22] Sommer F, Bäckhed F (2013). The gut microbiota—masters of host development and physiology. Nat Rev Microbiol.

[23] Ochsenkühn MA, Röthig T, D’Angelo C, Wiedenmann J, Voolstra CR (2017). The role of floridoside in osmoadaptation of coral-associated algal endosymbionts to high-salinity conditions. Sci Adv.

[24] Shaffer JP, U’Ren JM, Gallery RE, Baltrus DA, Arnold AE (2017). An endohyphal bacterium (Chitinophaga, Bacteroidetes) alters carbon source use by *Fusarium keratoplasticum* (*F. solani* species complex, Nectriaceae). Front Microbiol.

[25] Gould AL, Zhang V, Lamberti L, Jones EW, Obadia B, Korasidis N (2018). Microbiome interactions shape host fitness. Proc Natl Acad Sci.

[26] Ezenwa VO, Gerardo NM, Inouye DW, Medina M, Xavier JB (2012). Animal behavior and the microbiome. Science.

[27] Chilton SN, Enos MK, Burton JP, Reid G. The effects of diet and the microbiome on reproduction and longevity: a comparative review across 5 continents. J Nutr Food Sci. 2015;5(3).

[28] Jacob S, Parthuisot N, Vallat A, Ramon-Portugal F, Helfenstein F, Heeb P (2015). Microbiome affects egg carotenoid investment, nestling development and adult oxidative costs of reproduction in great tits. Funct Ecol.

[29] Weiland-Bräuer N, Pinnow N, Langfeldt D, Roik A, Güllert S, Chibani CM (2020). The native microbiome is crucial for offspring generation and fitness of *Aurelia aurita*. MBio.

[30] Bang C, Dagan T, Deines P, Dubilier N, Duschl WJ, Fraune S (2018). Metaorganisms in extreme environments: do microbes play a role in organismal adaptation?. Zoology.

[31] Torda G, Donelson JM, Aranda M, Barshis DJ, Bay L, Berumen ML (2017). Rapid adaptive responses to climate change in corals. Nat Clim Change.

[32] Ziegler M, Seneca FO, Yum LK, Palumbi SR, Voolstra CR (2017). Bacterial community dynamics are linked to patterns of coral heat tolerance. Nat Commun.

[33] Pita L, Rix L, Slaby BM, Franke A, Hentschel U (2018). The sponge holobiont in a changing ocean: from microbes to ecosystems. Microbiome.

[34] Voolstra CR, Ziegler M (2020). Adapting with microbial help: microbiome flexibility facilitates rapid responses to environmental change. BioEssays.

[35] Baldassarre L, Ying H, Reitzel AM, Franzenburg S, Fraune S (2022). Microbiota mediated plasticity promotes thermal adaptation in the sea anemone Nematostella vectensis. Nat Commun.

[36] Yampolsky LY, Schaer TM, Ebert D (2014). Adaptive phenotypic plasticity and local adaptation for temperature tolerance in freshwater zooplankton. Proc Royal Soc B: Biol Sci.

[37] Pazzaglia J, Reusch TB, Terlizzi A, Marín-Guirao L, Procaccini G (2021). Phenotypic plasticity under rapid global changes: the intrinsic force for future seagrasses survival. Evol Appl.

[38] Fagundes CT, Amaral FA, Teixeira AL, Souza DG, Teixeira MM (2012). Adapting to environmental stresses: the role of the microbiota in controlling innate immunity and behavioral responses. Immunol Rev.

[39] Koza NA, Adedayo AA, Babalola OO, Kappo AP (2022). Microorganisms in plant growth and development: roles in abiotic stress tolerance and secondary metabolites secretion. Microorganisms.

[40] Fang FC, Frawley ER, Tapscott T, Vázquez-Torres A (2016). Bacterial stress responses during host infection. Cell Host Microbe.

[41] Ost KS, Round JL (2018). Communication between the microbiota and mammalian immunity. Annu Rev Microbiol.

[42] Singh SK, Wu X, Shao C, Zhang H (2022). Microbial enhancement of plant nutrient acquisition. Stress Biology.

[43] Yin W, Wang Y, Liu L, He J, Biofilms (2019). The microbial protective clothing in extreme environments. Int J Mol Sci.

[44] Średnicka P, Juszczuk-Kubiak E, Wójcicki M, Akimowicz M, Roszko M (2021). Probiotics as a biological detoxification tool of food chemical contamination: a review. Food Chem Toxicol.

[45] Peixoto RS, Sweet M, Villela HD, Cardoso P, Thomas T, Voolstra CR (2021). Coral probiotics: premise, promise, prospects. Annu Rev Anim Biosci.

[46] Carrier TJ, Reitzel AM (2017). The hologenome across environments and the implications of a host-associated microbial repertoire. Front Microbiol.

[47] Rosenberg E, Zilber-Rosenberg I. The hologenome concept: human, animal and plant microbiota. Springer; 2014.

[48] Bordenstein SR, Theis KR (2015). Host biology in light of the microbiome: ten principles of holobionts and hologenomes. PLoS Biol.

[49] Röthig T, Ochsenkühn MA, Roik A, Van Der Merwe R, Voolstra CR (2016). Long-term salinity tolerance is accompanied by major restructuring of the coral bacterial microbiome. Mol Ecol.

[50] Ribes M, Calvo E, Movilla J, Logares R, Coma R, Pelejero C (2016). Restructuring of the sponge microbiome favors tolerance to ocean acidification. Environ Microbiol Rep.

[51] Ziegler M, Grupstra CG, Barreto MM, Eaton M, BaOmar J, Zubier K (2019). Coral bacterial community structure responds to environmental change in a host-specific manner. Nat Commun.

[52] Webster NS, Reusch TB (2017). Microbial contributions to the persistence of coral reefs. ISME J.

[53] Moran NA. Symbiosis as an adaptive process and source of phenotypic complexity. Proceedings of the National Academy of Sciences. 2007;104(suppl_1):8627-33. 10.1073/pnas.0611659104 PMC1876439 17494762

[54] Soen Y (2014). Environmental disruption of host–microbe co-adaptation as a potential driving force in evolution. Front Genet.

[55] O’Brien PA, Webster NS, Miller DJ, Bourne DG (2019). Host-microbe coevolution: applying evidence from model systems to complex marine invertebrate holobionts. MBio.

[56] Dong Z. Blooms of the moon jellyfish *Aurelia*: causes, consequences and controls. World Seas: an environmental evaluation. Elsevier; 2019. 163–71.

[57] Pikesley SK, Godley BJ, Ranger S, Richardson PB, Witt MJ (2014). Cnidaria in UK coastal waters: description of spatio-temporal patterns and inter-annual variability. J Mar Biol Association United Kingd.

[58] Gershwin L-A, Stung!. On jellyfish blooms and the future of the ocean. University of Chicago Press; 2013.

[59] Purcell JE, Uye S-i, Lo W-T (2007). Anthropogenic causes of jellyfish blooms and their direct consequences for humans: a review. Mar Ecol Prog Ser.

[60] Stoltenberg I, Dierking J, Müller-Navarra DC, Javidpour J (2021). Review of jellyfish trophic interactions in the Baltic Sea. Mar Biol Res.

[61] Brierley AS, Kingsford MJ (2009). Impacts of climate change on marine organisms and ecosystems. Curr Biol.

[62] Condon RH, Duarte CM, Pitt KA, Robinson KL, Lucas CH, Sutherland KR et al. Recurrent jellyfish blooms are a consequence of global oscillations. Proceedings of the National Academy of Sciences. 2013;110(3):1000-5. 10.1073/pnas.1210920110 PMC3549082 23277544

[63] Brotz L, Pauly D (2012). Jellyfish populations in the Mediterranean Sea. Acta Adriat.

[64] Henschke N, Stock CA, Sarmiento JL (2018). Modeling population dynamics of scyphozoan jellyfish (Aurelia spp.) in the Gulf of Mexico. Mar Ecol Prog Ser.

[65] Gibbons MJ, Richardson AJ. Patterns of jellyfish abundance in the North Atlantic. In: Jellyfish Blooms: Causes, Consequences, and Recent Advances: Proceedings of the Second International Jellyfish Blooms Symposium, held at the Gold Coast, Queensland, Australia, 24–27 June, 2007. Springer; 2009: 51–65.

[66] Richardson AJ, Bakun A, Hays GC, Gibbons MJ (2009). The jellyfish joyride: causes, consequences and management responses to a more gelatinous future. Trends Ecol Evol.

[67] Lucas CH (2001). Reproduction and life history strategies of the common jellyfish, *Aurelia aurita*, in relation to its ambient environment. Hydrobiologia.

[68] Pitt K, Kingsford M (2000). Reproductive biology of the edible jellyfish Catostylus mosaicus (Rhizostomeae). Mar Biol.

[69] Xing Y, Liu Q, Zhang M, Zhen Y, Mi T, Yu Z (2020). Effects of temperature and salinity on the asexual reproduction of Aurelia coerulea polyps. J Oceanol Limnol.

[70] Schäfer S, Gueroun SK, Andrade C, Canning-Clode J (2021). Combined Effects of temperature and salinity on polyps and Ephyrae of Aurelia solida (Cnidaria: Scyphozoa). Diversity.

[71] Bamstedt U (1990). Trophodynamics of the scyphomedusae *Aurelia aurita*. Predation rate in relation to abundance, size and type of prey organism. J Plankton Res.

[72] Widmer CL. Effects of temperature on growth of north-east Pacific moon jellyfish ephyrae, Aurelia. J Mar Biol Association United Kingd. 2005.

[73] Pascual M, Fuentes V, Canepa A, Atienza D, Gili JM, Purcell JE (2015). Temperature effects on asexual reproduction of the scyphozoan Aurelia aurita sl: differences between exotic (baltic and red seas) and native (Mediterranean Sea) populations. Mar Ecol.

[74] Widmer CL, Fox CJ, Brierley AS (2016). Effects of temperature and salinity on four species of northeastern Atlantic scyphistomae (Cnidaria: Scyphozoa). Mar Ecol Prog Ser.

[75] Willcox S, Moltschaniwskyj NA, Crawford C (2007). Asexual reproduction in scyphistomae of Aurelia sp.: Effects of temperature and salinity in an experimental study. J Exp Mar Biol Ecol.

[76] Purcell JE, Atienza D, Fuentes V, Olariaga A, Tilves U, Colahan C et al. Temperature effects on asexual reproduction rates of scyphozoan species from the northwest Mediterranean Sea. Jellyfish Blooms IV. Springer; 2012. 169–80.

[77] Loveridge A, Lucas CH, Pitt KA (2021). Shorter, warmer winters may inhibit production of ephyrae in a population of the moon jellyfish Aurelia aurita. Hydrobiologia.

[78] Conley K, Uye S-i (2015). Effects of hyposalinity on survival and settlement of moon jellyfish (Aurelia aurita) planulae. J Exp Mar Biol Ecol.

[79] Watanabe T, Ishii H (2001). *Situ* estimation of ephyrae liberated from polyps of *Aurelia aurita* using settling plates in Tokyo Bay, Japan. Hydrobiologia.

[80] Sokołowski A, Brulińska D, Olenycz M, Wołowicz M (2016). Does temperature and salinity limit asexual reproduction of Aurelia aurita polyps (Cnidaria: Scyphozoa) in the Gulf of Gdańsk (southern Baltic Sea)? An experimental study. Hydrobiologia.

[81] Janas U, Witek Z. The occurrence of medusae in the southern Baltic and their importance in the ecosystem, with special emphasis on Aurelia aurita. Oceanologia. 1993;(34).

[82] Goldstein J, Steiner UK. Ecological drivers of jellyfish blooms–the complex life history of a ‘well-known’medusa (*Aurelia aurita*). J Anim Ecol. 2019. 10.1111/1365-2656.13147 31782797

[83] Jensen N, Weiland-Bräuer N, Joel S, Chibani CM, Schmitz RA. Asexual reproduction of *Aurelia aurita* depends on the presence of a balanced microbiome at polyp stage. In.: Research Square; 2023. 10.1128/spectrum.00262-23 PMC10433978 37378516

[84] Weiland-Bräuer N, Neulinger SC, Pinnow N, Kunzel S, Baines JF, Schmitz RA (2015). Composition of bacterial Communities Associated with *Aurelia aurita* Changes with Compartment, Life Stage, and Population. Appl Environ Microbiol.

[85] Weiland-Bräuer N, Prasse D, Brauer A, Jaspers C, Reusch TBH, Schmitz RA (2020). Cultivable microbiota associated with *Aurelia aurita* and *Mnemiopsis leidyi*. MicrobiologyOpen.

[86] Gac J-P, Marrec P, Cariou T, Grosstefan E, Macé E, Rimmelin-Maury P et al. Decadal Dynamics of the CO2 system and Associated Ocean Acidification in Coastal Ecosystems of the North East Atlantic Ocean. Front Mar Sci. 2021:759.

[87] Lane BG, Bernier F, Dratewka-Kos E, Shafai R, Kennedy TD, Pyne C (1991). Homologies between members of the germin gene family in hexaploid wheat and similarities between these wheat germins and certain Physarum spherulins. J Biol Chem.

[88] Jo AR, Lee JY, Timmermann A, Jin FF, Yamaguchi R, Gallego A. Future amplification of Sea Surface temperature seasonality due to enhanced Ocean Stratification. Geophys Res Lett. 2022:e2022GL098607.

[89] Cheng L, Trenberth KE, Gruber N, Abraham JP, Fasullo JT, Li G (2020). Improved estimates of changes in upper ocean salinity and the hydrological cycle. J Clim.

[90] Miyama T, Minobe S, Goto H (2021). Marine heatwave of sea surface temperature of the Oyashio region in summer in 2010–2016. Front Mar Sci.

[91] Feudale L, Shukla J (2011). Influence of sea surface temperature on the european heat wave of 2003 summer. Part I: an observational study. Clim Dyn.

[92] Feudale L, Shukla J (2011). Influence of sea surface temperature on the european heat wave of 2003 summer. Part II: a modeling study. Clim Dyn.

[93] Klein JP, Goel P. Survival analysis: state of the art. 1992.

[94] Anderson MJ (2001). A new method for non-parametric multivariate analysis of variance. Austral Ecol.

[95] Dixon P (2003). VEGAN, a package of R functions for community ecology. J Veg Sci.

[96] Oksanen J, Blanchet FG, Kindt R, Legendre P, Minchin PR, O´Hara RB (2013). Package ´vegan´ R Packag ver.

[97] Schloss PD, Westcott SL, Ryabin T, Hall JR, Hartmann M, Hollister EB (2009). Introducing mothur: open-source, platform-independent, community-supported software for describing and comparing microbial communities. Appl Environ Microbiol.

[98] Hansen J, Sato M, Ruedy R. Global Temperature in 2021. Diponível em http://www columbia edu/~ jeh1/mailings/2022/Temperature2021 13January2022 pdf Acesso em. 2022;1(02).

[99] Oliver EC, Donat MG, Burrows MT, Moore PJ, Smale DA, Alexander LV (2018). Longer and more frequent marine heatwaves over the past century. Nat Commun.

[100] Von Kietzell A, Schurer A, Hegerl GC (2022). Marine heatwaves in global sea surface temperature records since 1850. Environ Res Lett.

[101] Smith KE, Burrows MT, Hobday AJ, King NG, Moore PJ, Sen Gupta A et al. Biological Impacts of Marine Heatwaves. Annual Rev Mar Sci. 2022;15. 10.1146/annurev-marine-032122-121437 35977411

[102] Durack PJ, Wijffels SE (2010). Fifty-year trends in global ocean salinities and their relationship to broad-scale warming. J Clim.

[103] Evans TG, Kültz D (2020). The cellular stress response in fish exposed to salinity fluctuations. J Experimental Zool Part A: Ecol Integr Physiol.

[104] Przeslawski R, Byrne M, Mellin C (2015). A review and meta-analysis of the effects of multiple abiotic stressors on marine embryos and larvae. Glob Change Biol.

[105] Velasco J, Gutiérrez-Cánovas C, Botella-Cruz M, Sánchez-Fernández D, Arribas P, Carbonell JA (2019). Effects of salinity changes on aquatic organisms in a multiple stressor context. Philosophical Trans Royal Soc B.

[106] Nahar L, Aycan M, Hanamata S, Baslam M, Mitsui T (2022). Impact of single and combined salinity and high-temperature stresses on agro-physiological, biochemical, and transcriptional responses in rice and stress-release. Plants.

[107] Toby Kiers E, Palmer TM, Ives AR, Bruno JF, Bronstein JL (2010). Mutualisms in a changing world: an evolutionary perspective. Ecol Lett.

[108] Ghaderiardakani F, Quartino ML, Wichard T (2020). Microbiome-dependent adaptation of seaweeds under environmental stresses: a perspective. Front Mar Sci.

[109] Herrera M, Klein SG, Schmidt-Roach S, Campana S, Cziesielski MJ, Chen JE (2020). Unfamiliar partnerships limit cnidarian holobiont acclimation to warming. Glob Change Biol.

[110] Randle JL, Cárdenas A, Gegner HM, Ziegler M, Voolstra CR (2020). Salinity-conveyed Thermotolerance in the Coral Model Aiptasia is accompanied by distinct changes of the bacterial microbiome. Front Mar Sci.

[111] Prazeres M, Ainsworth T, Roberts TE, Pandolfi JM, Leggat W (2017). Symbiosis and microbiome flexibility in calcifying benthic foraminifera of the great barrier reef. Microbiome.

[112] Santoro EP, Borges RM, Espinoza JL, Freire M, Messias CS, Villela HD (2021). Coral microbiome manipulation elicits metabolic and genetic restructuring to mitigate heat stress and evade mortality. Sci Adv.

[113] Rubio-Portillo E, Ramos-Esplá AA, Antón J (2021). Shifts in marine invertebrate bacterial assemblages associated with tissue necrosis during a heat wave. Coral Reefs.

[114] Marangon E, Laffy PW, Bourne DG, Webster NS (2021). Microbiome-mediated mechanisms contributing to the environmental tolerance of reef invertebrate species. Mar Biol.

[115] Wang M, Jeong C-B, Lee YH, Lee J-S (2018). Effects of ocean acidification on copepods. Aquat Toxicol.

[116] Leger RJS (2021). Insects and their pathogens in a changing climate. J Invertebr Pathol.

[117] Carballo JL, Bell JJ. Climate change and sponges: an introduction. Climate Change, Ocean Acidification and Sponges. Springer; 2017. 1–11.

[118] Gegner HM, Rädecker N, Ochsenkühn M, Barreto MM, Ziegler M, Reichert J (2019). High levels of floridoside at high salinity link osmoadaptation with bleaching susceptibility in the cnidarian-algal endosymbiosis. Biology Open.

[119] Webster N, Negri A, Botté E, Laffy P, Flores F, Noonan S (2016). Host-associated coral reef microbes respond to the cumulative pressures of ocean warming and ocean acidification. Sci Rep.

[120] Pootakham W, Mhuantong W, Yoocha T, Putchim L, Jomchai N, Sonthirod C (2019). Heat-induced shift in coral microbiome reveals several members of the Rhodobacteraceae family as indicator species for thermal stress in Porites lutea. MicrobiologyOpen.

[121] Dungan AM, Bulach D, Lin H, van Oppen MJ, Blackall LL (2021). Development of a free radical scavenging bacterial consortium to mitigate oxidative stress in cnidarians. Microb Biotechnol.

[122] Sabdono A, Sawonua PH, Kartika AGD, Amelia JM, Radjasa OK (2015). Coral diseases in Panjang Island, Java Sea: diversity of anti–pathogenic bacterial coral symbionts. Procedia Chem.

[123] Thompson JR, Rivera HE, Closek CJ, Medina M (2015). Microbes in the coral holobiont: partners through evolution, development, and ecological interactions. Front Cell Infect Microbiol.

[124] Tinta T, Kogovšek T, Klun K, Malej A, Herndl GJ, Turk V (2019). Jellyfish-associated microbiome in the marine environment: exploring its biotechnological potential. Mar Drugs.

[125] Berg G, Rybakova D, Fischer D, Cernava T, Vergès M-CC, Charles T (2020). Microbiome definition re-visited: old concepts and new challenges. Microbiome.

[126] Henry LP, Bruijning M, Forsberg SK, Ayroles JF (2021). The microbiome extends host evolutionary potential. Nat Commun.

