# Supplementary material for: Microbial community changes correlate with impaired host fitness of Aurelia aurita after environmental challenge
Source: Anim Microbiome. 2023 Sep 21;5:45. doi: 10.1186/s42523-023-00266-4 (PMC10515101; doi:10.1186/s42523-023-00266-4)
Supplement: Supplementary file 1 — Supplementary Material 1. Tables S1-S6: PERMANOVA tests.: S1-Survival, S2-Growth, S3-Feeding rates, S4-Budding, S5-Segmentation (strobilation), S6-Ephyrae release. Figure S1: Monitoring polyp fitness traits survival and growth. [file 42523_2023_266_MOESM1_ESM.pdf]

Microbial community changes correlate with impaired host fitness of *Aurelia aurita* after environmental challenge

Nicole Pinnow[1,#], Cynthia M. Chibani [1], Simon Güllert[1, †], and Nancy Weiland-Bräuer[1, #, *]

[1] Kiel University, General Microbiology, Am Botanischen Garten 1-9, 24118 Kiel, Germany

[#] Joint first authors

[†] Current address: Sysmex Inostics GmbH, Falkenried 88, 20251 Hamburg, Germany

[*] Correspondence: nweiland@ifam.uni-kiel.de; Tel.: , 49-431-880-1649 (Germany)

Keywords: metaorganism, *Aurelia aurita*, environment, climate change, microbiome, acclimatization, salinity, temperature, future ocean

Tab. S1: Pairwise result of Log-rank test for survival. Log-rank test was used with the survival library (https://cran.r-project.org/web/packages/survival/index.html). Kaplan Meier test produced % of survival rates, chi-squares, and calculated the listed p-values for significance (< 0.05). Significance levels *, < 0.05; **, < 0.01; ns, not significant.

fitness trait: survival

**comparison**		**p-value**	**significance**
native normal	sterile normal	0.300	ns
native normal	native high salinity	0.250	ns
native normal	native high temperature	0.003	**
native normal	native future ocean	0.400	ns
sterile normal	sterile high salinity	0.020	*
sterile normal	sterile high temperature	0.010	*
sterile normal	sterile future ocean	0.200	ns
native high salinity	sterile high salinity	0.030	*
native high temperature	sterile high temperature	0.600	ns
native future ocean	sterile future ocean	0.200	ns

Tabs. S2-S6: PERMANOVA tests. Multivariate statistics were conducted using R for each fitness trait and the treatments growth, feeding, budding, segmentation, and ephyrae release for native and sterile conditions. PERMANOVA p-values are depicted with their corresponding significance levels *, < 0.05; **, < 0.01; ***,< 0.001; ns, not significant. PERMANOVA settings included Permutation test for adonis under reduced model, free Permutation, and 9999 No. of permutations adonis2.

Tab. S2

fitness trait: growth

comparison		p-value	significance
native normal	sterile normal	0.001	***
native normal	native high salinity	0.001	***
native normal	native high temperature	0.001	***
native normal	native future ocean	0.001	***
sterile normal	sterile high salinity	0.001	***
sterile normal	sterile high temperature	0.001	***
sterile normal	sterile future ocean	0.002	**
native high salinity	sterile high salinity	0.001	***
native high temperature	sterile high temperature	0.012	*
native future ocean	sterile future ocean	0.042	*

Tab. S3

fitness trait: feeding

comparison		p-value	significance
native normal	sterile normal	0.896	ns
native normal	native high salinity	0.030	*
native normal	native high temperature	0.036	*
native normal	native future ocean	0.592	ns
sterile normal	sterile high salinity	0.010	**
sterile normal	sterile high temperature	0.010	**
sterile normal	sterile future ocean	0.016	*
native high salinity	sterile high salinity	0.596	ns
native high temperature	sterile high temperature	0.498	ns
native future ocean	sterile future ocean	0.110	ns

Tab. S4

fitness trait: budding

comparison		p-value	significance
native normal	sterile normal	0.001	***
native normal	native high salinity	0.001	***
native normal	native high temperature	0.001	***
native normal	native future ocean	0.001	***
sterile normal	sterile high salinity	0.001	***

sterile normal	sterile high temperature	0.001	***
sterile normal	sterile future ocean	0.001	***
native high salinity	sterile high salinity	0.775	ns
native high temperature	sterile high temperature	0.001	***
native future ocean	sterile future ocean	0.010	**

Tab. S5

fitness trait: segmentation

comparison		p-value	significance
native normal	sterile normal	0.001	***
native normal	native high salinity	0.001	***
native normal	native high temperature	0.001	***
native normal	native future ocean	0.001	***
sterile normal	sterile high salinity	0.001	***
sterile normal	sterile high temperature	0.001	***
sterile normal	sterile future ocean	0.001	***
native high salinity	sterile high salinity	0.002	**
native high temperature	sterile high temperature	0.001	***
native future ocean	sterile future ocean	0.007	**

Tab. S6

fitness trait: ephyrae release

comparison		p-value	significance
native normal	sterile normal	0.001	***
native normal	native high salinity	0.001	***
native normal	native high temperature	0.001	***
native normal	native future ocean	0.001	***
sterile normal	sterile high salinity	0.002	**
sterile normal	sterile high temperature	0.001	***
sterile normal	sterile future ocean	0.001	***
native high salinity	sterile high salinity	0.003	**
native high temperature	sterile high temperature	0.897	ns
native future ocean	sterile future ocean	0.002	**

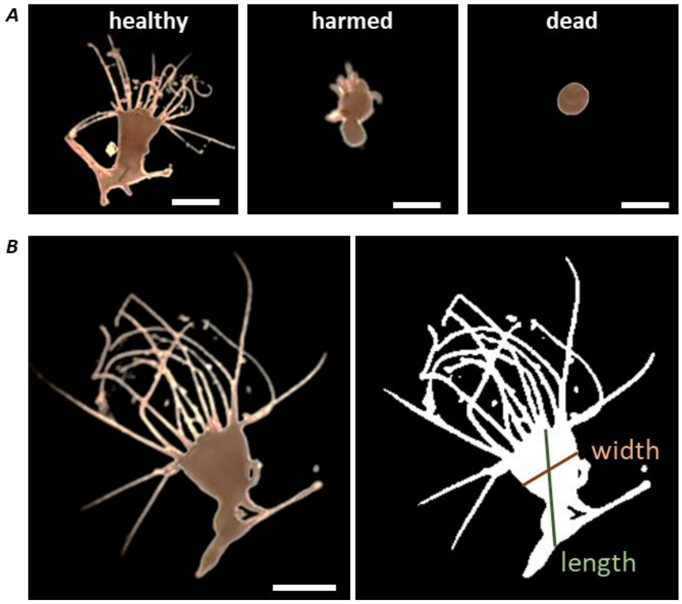

Fig. S1: Monitoring polyp fitness traits survival and growth. (***A***) Polyps categorized as healthy, harmed, and dead based on their phenotypical appearance. Healthy and harmed are alive polyps. (***B***) Example original photograph of an alive polyp (left panel) and its black/white projection (right panel) showing the axes for length and width measurement of the calyx.
